# The Atonal Proneural Transcription Factor Links Differentiation and Tumor Formation in *Drosophila*

**DOI:** 10.1371/journal.pbio.1000040

**Published:** 2009-02-24

**Authors:** Wouter Bossuyt, Natalie De Geest, Stein Aerts, Iris Leenaerts, Peter Marynen, Bassem A Hassan

**Affiliations:** 1 Laboratory of Neurogenetics, Department of Molecular and Developmental Genetics, VIB, Leuven, Belgium; 2 Department of Human Genetics, K.U. Leuven School of Medicine, Leuven, Belgium; 3 Doctoral Program in Molecular and Developmental Genetics, K.U. Leuven Group Biomedicine, Leuven, Belgium; 4 The Human Genome Laboratory, Department of Molecular and Developmental Genetics, VIB, Leuven, Belgium; Western General Hospital, United Kingdom

## Abstract

The acquisition of terminal cell fate and onset of differentiation are instructed by cell type–specific master control genes. Loss of differentiation is frequently observed during cancer progression, but the underlying causes and mechanisms remain poorly understood. We tested the hypothesis that master regulators of differentiation may be key regulators of tumor formation. Using loss- and gain-of-function analyses in *Drosophila*, we describe a critical anti-oncogenic function for the *atonal* transcription factor in the fly retina, where *atonal* instructs tissue differentiation. In the tumor context, *atonal* acts by regulating cell proliferation and death via the JNK stress response pathway. Combined with evidence that *atonal*'s mammalian homolog, *ATOH1*, is a tumor suppressor gene, our data support a critical, evolutionarily conserved, function for *ato* in oncogenesis.

## Author Summary

During embryonic development, cells become more and more specialized, and this process is referred to as differentiation. In contrast to normal adult cells, cancer cells—like embryonic cells—display fewer differentiated properties. It has been postulated that the acquisition of terminal differentiation helps inhibit tumor formation; however, no direct evidence for this hypothesis was available. The development of the eye in the fruit fly, Drosophila melanogaster, has long been used as a model for studying genetic factors controlling differentiation. More recently, eye development has also been used to study how tumors can form and progress. In this study, we used this model to show that genes, such as *atonal*, that instruct the differentiation of specific tissues can act as tumor suppressers and inhibit the formation and progression of tumors in those tissues. Losing such genes can generate tumors, whereas activating them can strongly inhibit these tumors.

## Abstract 3

We establish a direct genetic link between cancer and the initiation of differentiation in the*Drosophila* eye.

## Introduction

Cell fate commitment in neural and neuroendocrine lineages of the peripheral nervous system (PNS) as well as secretory epithelia is controlled by genes of the basic helix-loop-helix (bHLH) superfamily of transcription factors. One of the most structurally and functionally conserved groups within this family is the Atonal (Ato) group proteins [1,2]. Drosophila ato (CG7508) and mammalian *ATOH1* (Ensembl accession number: ENSG00000172238) are required for cell fate specification and the induction of differentiation in the PNS and the secretory lineages in all animal species. In *Drosophila*, *ato* is necessary for cell fate specification and differentiation of mechano- and photoreceptors [3–5].

The acquisition of differentiated cell fate endows cells with two key features. First, it allows them to become distinct from one another and, accordingly, functionally specialized. Second, it inhibits further cell division under physiological conditions, thus controlling tissue size. When the regulation of cell division fails, cancer may develop. Cancer, however, is the result of a selective process in which cells accumulate several genetic and epigenetic mutations giving them a growth advantage over surrounding cells by, for example, the inhibition of apoptosis, induction of angiogenesis, and growth factor–independent survival [6]. More than one mutation is needed for cancer to arise, and it is therefore thought that mutations occur in undifferentiated cells that are proliferative. As such, oncogenesis might select for cells that have lost their capacity to induce differentiation. In this context, it has been a long-standing postulate that cancer is a disease of loss of differentiation [7,8]. Work in the seventies and eighties by Harris and colleagues shows that hybrids of malignant and diploid cells only become malignant again after losing chromosomal loci required for differentiation (e.g., [9]). More recently, the interplay between differentiation and cancer has gained renewed attention through the study of a pool of undifferentiated cells in tumors, the so-called cancer stem or tumor-initiating cells [10]. A major theme emerging from these studies is the importance of the maintenance of an undifferentiated state in this niche for tumor growth to occur. Furthermore, the fact that signals implicated in regulating differentiation across various lineages, such as the WNT and Notch pathways, also have been implicated both in the promotion and suppression of cancer [11,12] suggests a mechanistic link between the regulation of differentiation and tumor progression. Importantly, however, these pathways are also implicated in stem cell or progenitor cell maintenance and do not act in a lineage-restricted fashion. As such, the definition of their role in tumor progression vis-a-vis differentiation is unclear. For this hypothesis to be correct, at least one key prediction should hold true: master control genes that instruct cell fate commitment in specific lineages should act as brakes on the oncogenic process, either by preventing uncontrolled proliferation or by inducing cell death when a differentiated state can no longer be maintained. Thus, we hypothesize that such master control genes suppress both tumor formation and progression.

To test this prediction in lineages in which *ato* is the key regulator of cell fate commitment, we asked two experimental questions. First, does *ato* loss of function contribute to tumor initiation or progression in tissues where *ato* instructs differentiation, such as the *Drosophila* retina? Second, can *ato* gain of function inhibit the formation or progression of these tumors? Finally, we examined the genetic pathway by which *ato* suppresses tumor formation.

We find that loss of *ato* strongly enhances the formation and progression of tumors in flies. Conversely, gain of *ato* function strongly inhibits tumor formation and metastasis. Finally, we describe a highly conserved anti-oncogenic genetic pathway that links *ato* activity to the stress sensor Jun N-terminal kinase (JNK) pathway. Combined with genetic and molecular evidence from mouse and human cancer models [13], these data support a key role for *ato* in a very early step of the oncogenic process and suggest that mutations in master control genes of cell fate commitment may be pivotal switches during tumorigenesis.

## Results

### Gain and Loss of *ato* Function Suppresses and Enhances Tumor Formation, Respectively, in a *Drosophila* Cancer Paradigm

We took advantage of the genetic power of Drosophila melanogaster to investigate whether the gain and loss of function of *Drosophila ato* suppresses and enhances tumor formation, respectively. *ato* instructs differentiation in the *Drosophila* eye [4]. We therefore turned to a well-established in vivo eye cancer model, namely “eyeful” flies, which has been used to study the mechanisms of Rb and the PTEN-AKT pathway in cancer [14,15]. The eyeful flies have activated Notch signaling in the developing eye due to overexpression of the Notch ligand Delta (Dl, CG3619), combined with overexpression of *lola* (CG12052) and *psq* (CG2368). Flies overexpressing only Dl, leading to an increase in eye size but no tumors, will henceforth be called “sensitized” flies.

**Figure 1:**
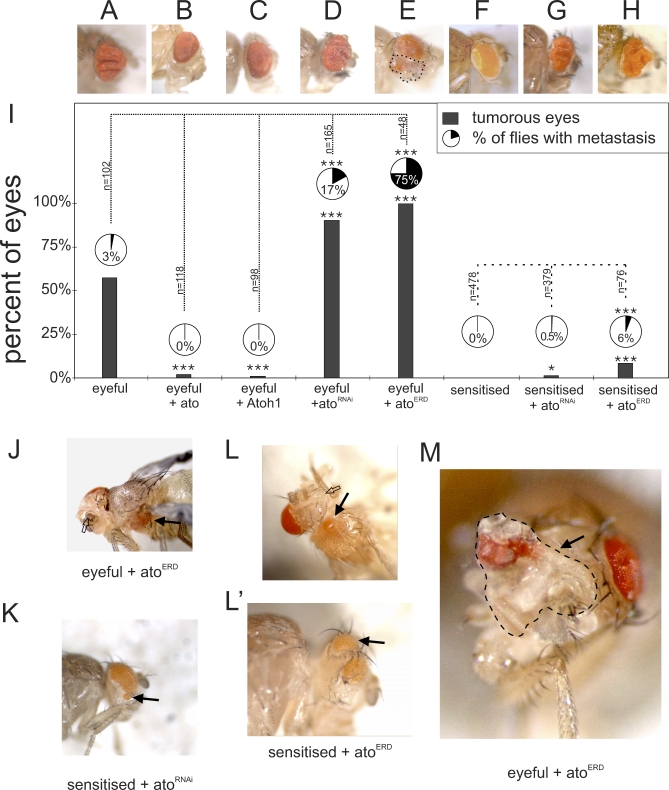
*atonal* Gain and Loss of Function Suppress and Promote Tumor Formation, Respectively, in a *Drosophila* Cancer Paradigm (A–I) Qualitative and quantitative representation of the tumor burden in different genotypes. Each lane is a separate genotype. Gray bars show percentage of tumorous eyes, pie charts show metastasis incidence. (A) *ey-GAL4, UAS-Dl, eyeful/^+^* fly (“^+^” = wild-type chromosome). (B and C) Gain of *ato* function suppresses tumor formation. (B) *ey-GAL4, UAS-Dl, eyeful/UAS-ato*. (C) *ey-GAL4, UAS-Dl, eyeful/UAS-Atoh1*. (D and E) Loss of *ato* function enhances tumor burden. (D) *ey-GAL4, UAS-Dl, eyeful/^+^; UAS-ato^RNAi^/^+^*. (E) *ey-GAL4, UAS-Dl, eyeful/UAS-ato^ERD^*. Dotted line indicates undifferentiated overgrowth of the eye tumor. (F) A sensitized genotype with eye-specific Dl overexpression leading to mild eye overgrowth: *ey-Gal4, UAS-Dl/^+^*. (G and H) Loss of *ato* function can initiate tumor formation. (G) *ey-Gal4, UAS-Dl/^+^, UAS-ato^RNAi^/^+^*. (H) *ey-Gal, UAS-Dl/UAS-ato^ERD^*. (I) Graph showing quantification of the tumor burden in the different genotypes. Dotted lines show the comparison used for statistical analysis. A single asterisk (*) indicates *p* < 0.05; triple asterisks (***) indicate *p* < 0.001 as analyzed by the chi-square test, and ‘n' represents number of flies analyzed. (J and K) Examples of metastasis and loss of differentiated eye tissue in eyeful and sensitized flies upon loss of *ato* function. (J) *ey-GAL4, UAS-Dl, eyeful/UAS-ato^ERD^*: black arrow shows metastasis, open arrow shows loss of differentiated eye tissue. (K) *ey-Gal4, UAS-Dl/^+^, UAS-ato^RNAi^/^+^* with metastasis on the head. (L and L′) *ey-Gal, UAS-Dl/UAS-ato^ERD^* showing metastasis in the thorax (black arrows) and on the head and loss of differentiated retina (open arrow). (M) *ey-Gal, UAS-Dl/UAS-ato^ERD^* showing a massive undifferentiated tumor (dotted line) on the fly head.

To analyze the tumor burden, each eye was scored separately. Eyes were counted as hyperplastic when the eye showed at least one fold. Metastasis can be seen as masses of amorphous red-pigmented cells outside of the eye field and are observed on the head and in the thorax and abdomen (Figure 1J–1L). Consistent with previous data, eyeful flies display excessively enlarged eyes, and eye tumors occur in 57% of the eyes, with 3% of the flies showing macroscopically visible metastases derived from the developing retina (*n* = 102; Figures 1A, 1I, S1A, and S1B). Overexpression of *ato*, or its mouse ortholog *Atoh1* (Ensembl: ENSMUSG00000073043)—but not a green fluorescent protein (GFP) control transgene—in the eyeful background almost completely suppresses the formation of eye tumors (Figure 1B (*ato*): 2%, *p* < 0.0001, *n* = 118; Figure 1C (*Atoh1*): 1%, *p* < 0.0001, *n* = 98; GFP (unpublished data): *p* = 0.25; Figure 1I). More importantly, reduction of endogenous *ato* expression in the eye using an *ato* RNA interference (RNAi) construct (a kind gift from A. Jarman), which leads to loss of differentiated eye tissue in wild-type flies (Figure S2B), results in a dramatic increase in both tumor incidence (90%, *p* < 0.0001, *n* = 165; Figure 1D and 1I) and the number of flies with metastases (17%, *p* = 0.0003). These effects were not due to the overexpression of a double-stranded RNA, per se, because the expression of an RNAi construct for GFP did not change the tumor burden (unpublished data; tumors: *p* = 0.59, metastasis: *p* = 0.21).

Since Ato is known as a transcriptional activator, we asked whether its role in eye tumors is mediated by its ability to activate gene expression. We constructed a repressor form of Ato by fusing it to an Engrailed repressor domain (Ato^ERD^) [16]. Expression of Ato^ERD^ in the developing eye leads to loss of differentiated eye tissue mimicking *ato* loss-of-function mutations and the *ato^RNAi^* construct (Figure S2A–S2C). Expression of Ato^ERD^ in eyeful flies results in both the loss of differentiated eye tissue in 30% of the eye fields (Figure 1J, open arrow), as well as in 100% tumors in the remaining eyes (*p* < 0.0001, *n* = 48; Figure 1E and 1I). Importantly, these tumors include large patches of undifferentiated tissue, showing that loss of *ato*'s differentiation function is linked to its anti-oncogenic function (Figure 1E and 1M, dotted line). Expression of Ato^ERD^ in the eyeful flies also results in 75% of the flies showing metastasis (Figure 1J, black arrow, and Figure 1I; *p* < 0.0001). These data suggest that *ato* is a key regulator of tumor progression in *Drosophila* and that it may perform this function by regulating the differentiation status of the transformed tissue.

### Ato Acts as a Switch for Tumor Initiation

Loss of *ato* in a wild-type background abrogates retinal differentiation and causes subsequent loss of the entire tissue [4]. If loss of differentiation is an early causal event in cancer, a key anti-oncogenic role for *ato* requires that its loss act as a switch for tumor initiation in a pre-oncogenic background. To this end, we used the sensitized genetic background that was used to generate the eyeful model, namely eye-specific Dl overexpression [14]. This genotype results in an increase in proliferation, leading in turn to a slight overgrowth of the eye, but no tumors are observed (*n* = 478; Figure 1F) [17]. Inhibition of *ato* function by Ato^ERD^ leads, as it does in wild-type flies, to loss of retinal differentiation (36% of the eye fields, empty arrow, Figure 1L), and to a 9% de novo tumor incidence in the remaining eye fields (*p* < 0.0001, *n* = 76; Figure 1H, 1L, and 1L′), with 6% of the flies showing metastasis (*p* = 0.0003). Similarly, *ato* knockdown using *ato^RNAi^* in this sensitized background leads to eye tumors in 0.5% of the eyes (*p* = 0.0333, *n* = 379; Figure 1G and 1K), and 0.3% of the flies have metastasis (*p* = 0.1953). The metastases in the sensitized flies upon loss of *ato* function are mostly present in the thorax and on the head. The metastases on the head (ato^RNAi^ = 75%; ato^ERD^ = 43%) show a high resemblance to ectopically induced eyes as seen by Kurata and colleagues upon overactivation of Notch signaling [18]. The loss of *ato* might increase Notch signaling and as such interfere with patterning and determination. We note, however, that we never see any head metastasis when overexpressing Dl, indicating that loss of *ato* creates new phenotypes including the metastasis in the thorax, which cannot be explained by an increase in Notch activity alone. In either case, these data support the hypothesis that loss of *ato* function is sufficient to transform a sensitized lesion into a metastatic tumor, possibly by interfering with patterning and determination.

### Ato Regulates Apoptosis and Proliferation in *Drosophila* Eye Tumors

**Figure 2:**
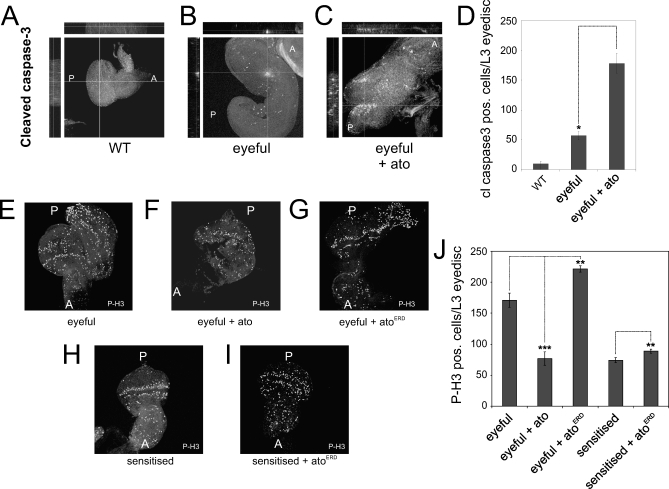
Apoptosis and Proliferation upon *ato* Loss and Gain of Function (A–C) Staining for cleaved caspase-3 indicates an increase in apoptosis upon overexpression of *ato* in the eyeful flies. (A) Immunohistochemistry for cleaved capsase-3 in wild type. (B) Cleaved caspase-3 staining in *ey-GAL4, UAS-Dl, eyeful/^+^.* (C) *ey-GAL4, UAS-Dl, eyeful/UAS-ato*. (D) Quantification of cleaved caspase-3–positive cells per eye disc. Error bars indicate the standard error of the mean, and double asterisks (**) indicate *p* < 0.05. (E–I) Representative images from third instar eye discs in different genetic backgrounds stained for phospho-Histone H3 (P-H3). (E) *ey-GAL4, UAS-Dl, eyeful/^+^*. (F) *ey-GAL4, UAS-Dl, eyeful/UAS-ato*. (G) *ey-GAL4, UAS-Dl, eyeful/UAS-ato^ERD^*. (H) *ey-GAL4, UAS-Dl/^+^*. (I) *ey-GAL4, UAS-Dl/UAS-ato^ERD^*. (J) Quantification of proliferating cells in the eyeful and sensitized background upon loss or gain of *ato* function. Quantification was done for a minimum of ten eye discs per genotype. The chi-square test was used to analyze for significance. Dotted lines connect compared genotypes. Triple asterisks (***) indicate *p* < 0.001; double asterisks (**) indicate *p* < 0.01.

*ato* loss- and gain-of-function analyses suggest a decisive role in tumor formation in the fly retina. The growth of tumors is a balance between cell proliferation and cell death. We asked whether *ato* regulates either or both of these processes in the context of eyeful tumors, during the development of these tumors. To this end, we examined third instar larval eye discs, the *Drosophila* eye anlage, for markers of apoptosis and proliferation. Overexpression of Ato in the eyeful fly results in dramatically increased levels of the apoptotic regulator caspase-3 (FlyBase ID: FBgn0028381) in eyeful eye discs (3-fold, *p* = 0.011; Figure 2A–2D). This explains, at least in part, the suppression of the eyeful tumors in the adult flies.

Next, we examined proliferation in eyeful eye discs under gain and loss of *ato* function conditions, using phospho-HistoneH3 (FlyBase ID:FBtr0071345) as a marker. Proliferation in the third instar eye disc normally occurs anterior to the morphogenetic furrow, where all the cells are still undifferentiated. Additionally, approximately two rows of undifferentiated cells posterior to the furrow, called the second mitotic wave (SMW), also proliferate. In the eyeful discs, proliferating cells are not restricted to these two domains but are also present posterior to the SMW (Figure 2E). Expression of *ato* in the eyeful disc reduces this ectopic proliferation (Figure 2F), whereas inhibition of *ato* activity increases ectopic proliferation (Figure 2G). As suppression of Ato activity can initiate de novo tumor formation in a sensitized background, we examined proliferation upon expression of Ato^ERD^ in the Dl-sensitized background. In the sensitized eye discs, proliferating cells are mostly restricted anterior to the furrow and the SMW (Figure 2H), whereas loss of *ato* function leads to the appearance of proliferating cells in the posterior region of the disc (Figure 2I).

The total number of cell divisions in a tissue determines the overall size of that tissue. We therefore quantified the total number of phospho-HistoneH3–positive cells per disc. Overexpression of Ato in eyeful eye discs results in a significant decrease in number of cells expressing the mitotic marker phosphorylated HistoneH3 (*p* = 0.00004; Figure 2J). Conversely, expression of the dominant-negative Ato^ERD^ leads to a significant up-regulation of proliferation in the eyeful eye discs (*p* = 0.004; Figure 2J). Thus, Ato limits number of cell divisions in the eyeful tumors. Expression of Ato^ERD^ in the Dl-sensitized eye discs results in a significant increase in phosphorylated HistoneH3 expression in the developing eye discs (*p* = 0.002; Figure 2J), explaining the induction of tumors by loss of *ato*.

Our analysis suggests that *ato* regulates both proliferation and death of retinal precursors during tumor formation in the *Drosophila* eye.

### Ato Regulates Tissue Differentiation and Patterning in *Drosophila* Eye Tumors

During normal development, Ato is required for the correct differentiation of retinal cells and the proper patterning of the eye disc. If Ato's function in suppressing eye tumors is related to its activity as a differentiation factor, we might expect to observe Ato-dependent alterations in tissue differentiation and organization upon manipulation of Ato activity in a tumor context. To test this prediction, we examined the expression of the early differentiation and R8 marker Senseless (Sens, CG32120), the general retinal photoreceptor marker embryonic lethal, abnormal vision (ELAV, CG4262), and the epithelial marker Armadillo/β-Catenin (Arm, CG11579) following manipulation of Ato activity.

**Figure 3:**
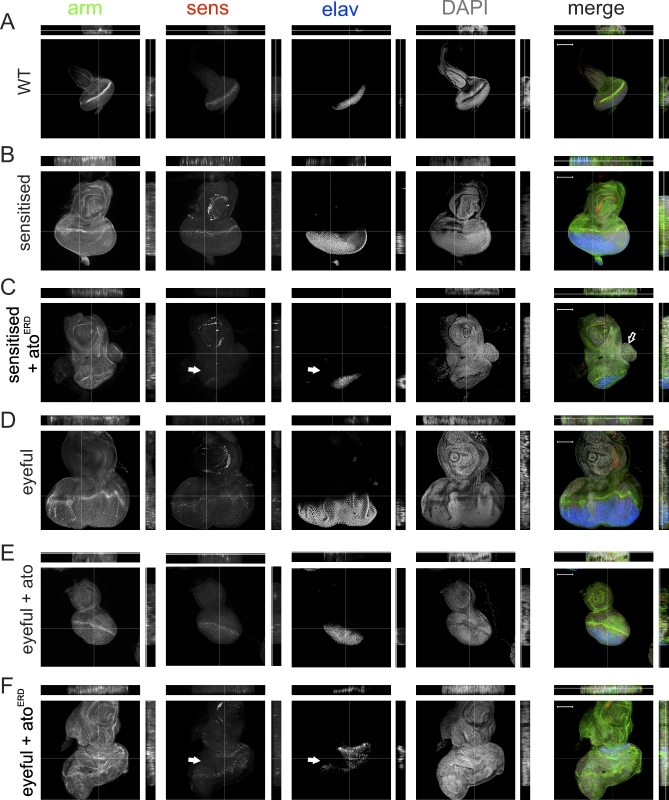
Analysis of Tissue Patterning and Differentiation in Third Instar Eye Discs Immunohistochemistry was used for armadillo (arm, green), embryonic lethal, abnormal vision (elav, blue), senseless (sens, red), and diamidinophenylindole (dapi, grey) (A) Wild-type eye disc. (B) *ey-GAL4, UAS-Dl/^+^* shows enlarged discs with wild-type patterning. (C) *ey-GAL4, UAS-Dl/UAS-ato^ERD^* show disrupted patterning with expansion of the undifferentiated domain (white arrows). Proliferative outgrowth is indicated with an open arrow. (D) *ey-GAL4, UAS-Dl, eyeful/^+^*. (E) Gain of *ato* function in the eyeful background leads to restoration of the pattern of differentiation: *ey-GAL4, UAS-Dl, eyeful/UAS-ato* shows almost normal appearance of all markers. (F) Loss of *ato* function in an eyeful background leads to loss of uniform arm staining and a loss of and abnormal pattern of differentiation (elav and sens, white arrows). *ey-GAL4, UAS-Dl, eyeful/UAS-ato^ERD^*. Images and orthogonal sections are shown. All images were taken at the depth of the nuclei. Scale bars represent 100 μm.

In wild-type and Dl-sensitized eye discs Arm, ELAV, and Sens reveal the regular and stereotypical differentiation and epithelial organization of the developing retina, although the Dl-sensitized discs are clearly larger (Figure 3A and 3B). Loss of Ato activity in the Dl-sensitized eye discs (Figure 3C) results in the disruption of the regular pattern of Arm expression, suggesting defects in the organization of the retinal epithelium. This is accompanied by severe reduction in Sens and ELAV staining, suggesting lack of differentiated photoreceptors. The proportion of undifferentiated to differentiated cells is increased, indicating that the initial steps of retinal differentiation, namely the specification of the Ato-dependent R8 cell, are compromised (Figure 3C, white arrows). In some instances, lobes of proliferative and undifferentiated tissue are observed in these eye discs (Figure 3C, open arrow), correlating with the appearance of tumors in the adult flies. In the eyeful eye discs, disorganization of the epithelium as well as defects in the pattern of differentiated cells are apparent (Figure 3D). Overexpression of Ato in the eyeful eye discs restores both the size and all three markers to essentially wild-type patterns of expression (Figure 3E), explaining the appearance of normal adult eyes in this background. Conversely, expression of Ato^ERD^ severely disrupts retinal patterning and the expression pattern of all three markers (Figure 3F). Differentiation markers are not only reduced, but also appear in a highly disruptive pattern to the extent that the morphogenetic furrow is difficult to discriminate (Figure 3F).

In summary, loss- and gain-of-function analyses in *Drosophila* support a critical and early role for the loss of *ato* in tumor initiation and progression. This effect is likely mediated by alteration in the expression of downstream genes required for retinal differentiation, as such perturbing proliferation, apoptosis, and tissue organization.

### Ato Functions via JNK-Dependent Mechanism

**Figure 4:**
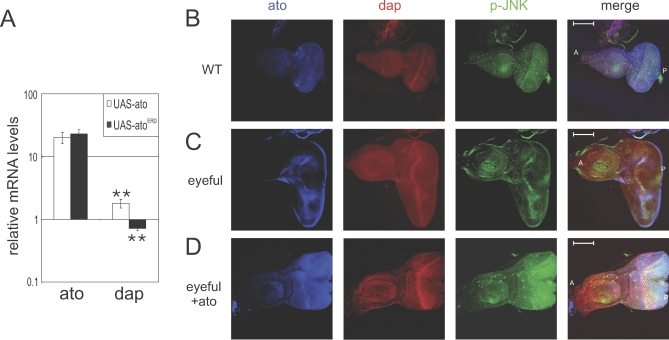
Ato Up-Regulates Dap and p-JNK (A) Quantitative RT-PCR for Ato (first two bars) and Dap (second two bars) upon Ato (white bars) and Ato^ERD^ (gray bars) expression, standardized to wild type and driver controls and to expression of three housekeeping genes. Error bars indicate the standard error of the mean. Double asterisks (**) indicate *p* < 0.01 (*t*-test). (B–D) Expression of *ato* in *ey-GAL4, UAS-Dl, eyeful* flies up-regulates Dap and phosphorylated JNK. (B) Third instar eye disc from wild type. (C) Third instar eye disc from *ey-GAL4, UAS-Dl, eyeful/^+^*. (D) Third instar eye discs from *ey-GAL4, UAS-Dl, eyeful/UAS-ato*. A indicates anterior; P indicates posterior.

To better understand the role of that Ato plays in tumor formation, we sought to determine the genetic mechanism by which it acts to suppress the formation and progression of tumors. Gain- and loss-of-function analysis indicated an *ato*-dependent regulation of proliferation in the *Drosophila* eye. Recently, the *Drosophila* ortholog of the gene encoding the cell cycle inhibitor p21^waf1^, *dacapo* (*dap*, CG1772), was reported to be a target gene of *ato* in the eye [19]. Consistent with this, overexpression of wild-type *ato* in the eye disc leads to significant up-regulation of *Dap* mRNA (∼80%, *p* = 0.018), whereas expression of Ato^ERD^ leads to significant down-regulation of *Dap* mRNA (28%, *p* = 0.014; Figure 4A). Ato expression also results in earlier onset and elevated Dap levels in the eyeful and wild-type eye discs, in agreement with the reduction in pH3 levels observed in the same discs (Figures 4B–4D and S3).

We have shown that Ato regulates apoptosis and that it restores proper differentiation in the eyeful eye discs. We reasoned that tumorous eyeful cells may interpret the Ato differentiation signal as cellular stress and, as a result, commit suicide. A major regulator of cell death in response to stress is the JNK pathway. We therefore examined the expression of phosphorylated (i.e., activated) form of the *Drosophila* JNK (pJNK), Basket (Bsk, CG5680). Eyeful discs show reduced pJNK levels. Overexpression of Ato in this background, as well as wild-type eye discs, results in dramatic up-regulation of pJNK levels (Figures 4B–4D and S3).

**Figure 5:**
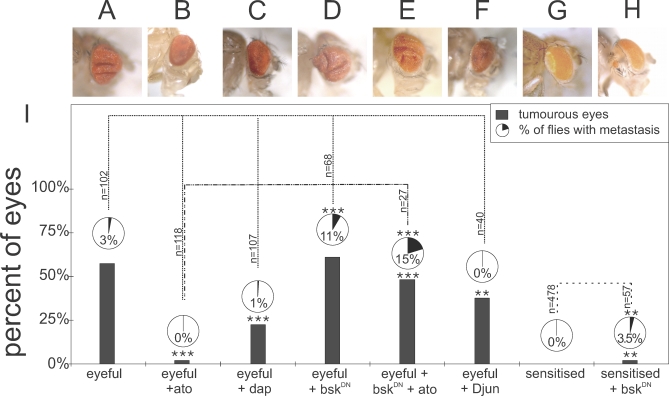
*ato* Functions by a JNK-Dependent Mechanism (A) *ey-GAL4, UAS-Dl, eyeful/^+^* fly. (B) *ey-GAL4, UAS-Dl, eyeful/UAS-ato.* (C) *ey-GAL4, UAS-Dl, eyeful/UAS-dap*. (D) *bsk^DN^/X; ey-GAL4, UAS-Dl, eyeful/^+^*. (E) *ato* function depends on JNK activity: *bsk^DN^/X; ey-GAL4, UAS-Dl, eyeful/UAS-ato*. (F) *ey-GAL4, UAS-Dl, eyeful/UAS-Djun*. (G) A sensitized genotype with eye-specific Dl overexpression leading to mild eye overgrowth: *ey-Gal4, UAS-Dl/^+^*. (H) *bsk^DN^/X; ey-GAL4, UAS-Dl/^+^*. (I) Quantitative representation of the tumor burden in different genotypes. Each lane is a separate genotype. Gray bars show percentage of tumorous eyes; pie charts give metastasis incidence. Double asterisks (**) indicate *p* < 0.01; triple asterisks (***) indicate *p* < 0.001 as analyzed by chi-square test, and n represents number of flies analyzed.

These data suggest that Ato regulates the expression and activity of major regulators of cell proliferation and death. We therefore tested whether these genes also play a role in the eyeful tumors. Dap overexpression leads to a significant inhibition of tumor occurrence (22%, *p* < 0.0001, *n* = 107; Figure 5C and 5I), but only a partial reduction in metastasis (1%, *p* = 0.1145). Thus, whereas Dap regulation appears to contribute to tumor suppression by Ato, it is unlikely to explain the full effect of *ato* expression.

To analyze whether the elevated activity of JNK signaling upon *ato* expression is functionally relevant, we inhibited JNK signaling using a dominant-negative form of Bsk (Bsk^DN^). This partially mimics down-regulation of *ato* in the eyeful model and results in tumors in 61% of the eyes (*p* = 0.574; Figure 5D and 5I) and an approximately7-fold increase in metastasis (*p* = 0.0003). Furthermore, expression of Bsk^DN^ in the Dl-sensitized background leads to the induction of tumors (2%, *p* = 0.0113, *n* = 57; Figure 5H and 5I) and metastasis (3.5%, *p* = 0.0112). Conversely, overexpression of *Djun* (Jra, CG2275), the transcriptional effector of the JNK pathway, leads to reduction of the tumor burden (38% tumors, *p* = 0.0036, *n* = 40; 0% metastasis, *p* = 0.559; Figure 5F and 5I), partially mimicking overexpression of Ato expression.

Next, we tested genetic epistasis between *ato* and JNK by overexpressing Ato while simultaneously inhibiting JNK signaling. This leads to a suppression of the inhibitory effects of *ato* on the eyeful flies and restores tumor formation (48%, *p* < 0.001), as well as enhances the metastatic phenotype (15%, *p* = 0.001, *n* = 27; Figure 5E and 5I). This indicates that JNK signaling is downstream of *ato* and that *ato* requires active JNK signaling to inhibit cancer formation.

## Discussion

Our data support a function for *ato* in oncogenesis. Loss of *ato* promotes tumor formation and progression and might, as such, be selected for during oncogenesis. This indicates that tumor formation and progression might not only require maintenance of self-renewal capacity, but also loss of the capacity to induce cell fate commitment and differentiation. Therefore, genes that act precisely at the junction of the transition from a proliferating progenitor to a committed cell ought to show anti-oncogenic behavior. Losing *ato* in the absence of any other compounding factor is neutral towards tumor formation. However, loss of *ato* in a sensitized background is sufficient to initiate and enhance tumor formation. In our experiments, we used activation of the Notch signaling pathway as a sensitizing factor, but other pathways also lead to the formation of tumors when *ato* is lost [13]. Therefore, loss of differentiation factors might “tip the balance” towards malignancy, regardless of what the additional oncogenic event may be. It will be interesting to investigate what the different pathways are that interact with loss of *ato* to enhance cancer formation and how they switch an *ato* mutation from neutral to tumor progression to oncogenic.

The induction of cellular differentiation acts on two levels: first, the cell cycle is inhibited by the expression of cell cycle inhibitors; and second, gene expression is modulated to instruct a specific fate and function. Several lines of evidence suggest that both levels of *ato* activity are important in its anti-oncogenic function. First, *ato* regulates the expression of *dap*—itself a direct target gene of *ato* during normal differentiation—during eye tumor formation. Second, loss of *ato* leads to more proliferation in the sensitized and cancerous tissue in a *Drosophila* model. Third, loss of *ato* leads to the disruption of retinal differentiation and patterning, correlating with the formation of tumors that include overgrowth of undifferentiated tissue in the fly eye. Together, these data support the idea that Ato exerts its anti-oncogenic function by activation of its developmental target genes and pathways. Finally, earlier reports suggest that, under certain conditions, proliferation can be uncoupled from the induction of differentiation as double-mutant cells for *retinoblastoma* and *dacapo* in the developing *Drosophila* eye keep proliferating although they start to differentiate [20]. Our data suggest that the inhibition of proliferation is not the only mechanism by which differentiation factors might suppress tumor formation, as *ato* is also able to induce apoptosis in an eyeful eye disc.

The function of JNK in the *Drosophila* eye has been described as both tumor promoting and anti-oncogenic. Igaki and colleagues describe a role for JNK in invasion upon loss of cell polarity [21], and Uhlirova et al. describe how JNK cooperates with Ras to induce tumor overgrowth in the eye [22]. In the overgrowth-sensitized setting of *scribble* mutant cells, however, JNK is necessary to remove these cells by apoptosis [22]. This shows that the molecular environment in which JNK acts decides the outcome. We propose that the status of differentiation might be an important factor in the decision of the outcome of JNK activity. *ato* function might divert JNK from an oncogenic function to a tumor suppressor function in which JNK will reduce the size of the overgrowth and, as such, reduce the number of metastases. Our data indicate that although JNK is necessary for the anti-oncogenic function of Ato, it is not sufficient, because inhibition of JNK signaling does not completely mimic the loss of *ato* function in the eyeful flies. This suggests JNK as a permissive, rather than instructive, factor for *ato*'s function and indicates that *ato* might also modulate tumor formation by JNK-independent mechanisms.

In summary, we present the first evidence that a master regulator of tissue-specific differentiation is a key regulator of tumor initiation and progression. The evidence that the human ortholog of Ato is a tumor suppressor gene in colorectal cancer, the largest cause of cancer deaths world-wide [13], as well as the absolute functional conservation between fly and mouse Ato [23] underscore the importance of understanding the fundamental molecular and genetic mechanisms of the function of this group of key developmental regulators.

## Materials and Methods

### *Drosophila* husbandry.

Fly strains used were *ey-GAL4, GS88A8, UAS-Dl/Cyo* (called eyeful flies in the text) and *ey-GAL4, UAS-Dl/Cyo* flies (a gift from M. Domiguez), *UAS-ato^RNAi^3B* and *UAS-ato^RNAi^ 4E* (gift from A. P. Jarman), *UAS-ato, UAS-Atoh1, w1118 P{UAS-bsk.DN}2, UAS-Djun, UAS-dacapo* (a gift from A. Hidalgo), *CantonS*, and *yw*. All flies were raised at 25 °C on standard fly food.

### Immunohistochemistry.

Eye discs of wandering third instar larva were dissected and processed as described [24]. *ato* antibody (kind gift from A. Jarman and P. zur Lage), Dap antibody (Developmental Studies Hybridoma Bank), P-JNK (Cell Signaling Technologies), phospho-HistoneH3 (Upstate Biotechnologies), and cleaved caspase-3 (Cell Signaling Technology).

### Generation of *UAS-ato^ERD^* transgenic flies.

*Uas-ato^ERD^* was generated by fusing the full-length Atonal ORF to a fragment encoding a Myc-tagged Engrailed repression domain (amino acids 2–298) [25] using the pUAST vector [26]. Seven *uas-ato^ERD^* transgenic lines were obtained using standard *Drosophila* transformation protocols.

### Quantitative reverse-transcriptase PCR on *Drosophila* larval eye-antennal discs.

Crosses between *ato-GAL4 (P{GawB}NP6558* obtained from *Drosophila* Genetic Resource Center, Kyoto) or *Gal4–7* and *UAS-ato* or *UAS-ato^ERD^* were performed at 18 °C and shifted to 28 °C at third larval instar stage. Eye-antennal discs were dissected in RNA later (Ambion). RNA extraction was performed with Mini RNA Isolation kit (Zymo Research). *act79B*, *gadph*, and *Rpl32* were used as control housekeeping genes (ΔCT), and *Canton S* and *UAS-Ato* as control RNA (ΔΔCT).

### Image quantification of proliferating and apoptotic cells.

The number of proliferating cells per eye disc was quantified using the “analyse particle” function in ImageJ with the parameters 5 to 60 for size and 0.5 to 1.0 for circularity.

## Supporting Information

Figure S1 Outgrowth Originates from the Eye Disc Proper (A) Third instar eye disc of *ey-Gal4>Dl>eyeful/^+^*. Confocal section of antibody-stained eye disc for senseless (red; indicating R8 photoreceptors), armadillo (green; indicating cell cortexes), and elav (blue; marker of mature neurons). Respective *z*-stacks are indicated next to the main image. White line indicates normal outline of eye-antennal imaginal disc. The malignant outgrowth (striped square) is enlarged in (B).
(B) Enlarged image from (A). Respective *z*-stacks are indicated next to the main image. White arrowhead indicates morphogenetic furrow in eye disc outgrowth. The different cell types present in a normal eye are also present in the outgrowth, indicating that the outgrowth originates from undifferentiated normal eye disc tissue.
(4.60 MB PDF)

Figure S2 Adult Loss of Phenotypes of Loss of *ato* Function (A) Representative picture *ey-Gal4*.
(B) Representative picture of *UAS-ato^RNAi^* driven by *ey-Gal4*. *ato^RNAi^* construct is active since the expression in the developing eye leads to a decrease in eye size.
(C) Representative picture of *UAS-ato^ERD^* driven by *ey-Gal4*. The *ato^ERD^* leads to a phenocopy of the loss of *ato*.
(378 KB PDF)

Figure S3 Ato, Dap, and pJNK Expression in Wild-Type Eye Discs Expression of *ato* in wild-type flies up-regulates Dap and phosphorylated JNK. Third instar eye disc from *eyeless-Gal4/UAS-ato* is shown.
(2.06 MB PDF)

## Abbreviations

Arm: Armadillo
Ato: Atonal
Bsk: Basket
Dl: Delta
ELAV: embryonic lethal, abnormal vision
JNK: Jun N-terminal kinase
pJNK: phosphorylated Jun N-terminal kinase
RNAi: RNA interference
Sens: Senseless
