# Supplementary material for: The Atonal Proneural Transcription Factor Links Differentiation and Tumor Formation in Drosophila
Source: PLoS Biol. 2009 Feb 24;7(2):e1000040. doi: 10.1371/journal.pbio.1000040 (PMC2652389; doi:10.1371/journal.pbio.1000040)
Supplement: Figure S1 — (A) Third instar eye disc of ey-Gal4>Dl>eyeful/+. Confocal section of antibody-stained eye disc for senseless (red; indicating R8 photoreceptors), armadillo (green; indicating cell cortexes), and elav (blue; marker of mature neurons). Respective z-stacks are indicated next to the main image. White line indicates normal outline of eye-antennal imaginal disc. The malignant outgrowth (striped square) is enlarged in (B). (B) Enlarged image from (A). Respective z-stacks are indicated next to the main image. White arrowhead indicates morphogenetic furrow in eye disc outgrowth. The different cell types present in a normal eye are also present in the outgrowth, indicating that the outgrowth originates from undifferentiated normal eye disc tissue. (4.60 MB PDF) [file pbio.1000040.sg001.pdf]

The Atonal proneural transcription factor links differentiation and tumor formation in *Drosophila*

Bossuyt *et al.*

Supplementary figures

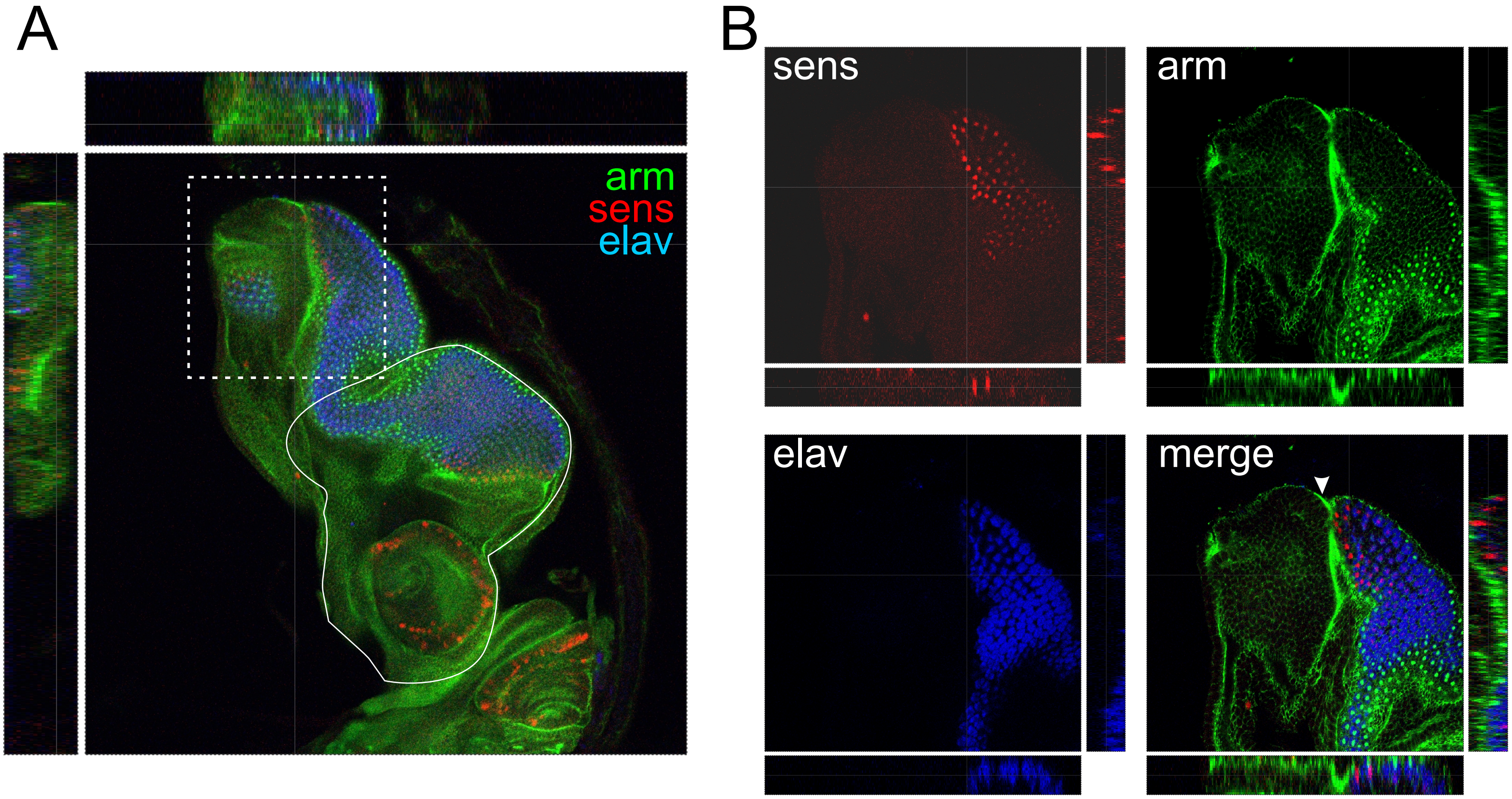

Supplementary Figure 1: Outgrowth originates form the eye disc proper. A, Third instar eyedisc of *ey-Gal4>Dl>eyeful/+*. Confocal section of antibody stained eye disc for senseless (red, indicating R8 photoreceptors), armadillo (green, indicating cell cortexes) and elav (bleu, marker of mature neurons). Respective z-stacks are indicated next to the main image. White line indicates normal outline of eye-antennal imaginal disc. The malignant outgrowth (striped square) is enlarged in b. **B,** Enlarged image from a. Respective z-stacks are indicated next to the main image. White arrow head indicates morphogenetic furrow in eye disc outgrowth. The different cell types present in a normal eye are also present in the outgrowth indicating that the outgrowth originates from undifferentiated normal eye disc tissue.
