# Supplementary material for: The Atonal Proneural Transcription Factor Links Differentiation and Tumor Formation in Drosophila
Source: PLoS Biol. 2009 Feb 24;7(2):e1000040. doi: 10.1371/journal.pbio.1000040 (PMC2652389; doi:10.1371/journal.pbio.1000040)
Supplement: Figure S2 — (A) Representative picture ey-Gal4. (B) Representative picture of UAS-atoRNAi driven by ey-Gal4. atoRNAi construct is active since the expression in the developing eye leads to a decrease in eye size. (C) Representative picture of UAS-atoERD driven by ey-Gal4. The atoERD leads to a phenocopy of the loss of ato. (378 KB PDF) [file pbio.1000040.sg002.pdf]

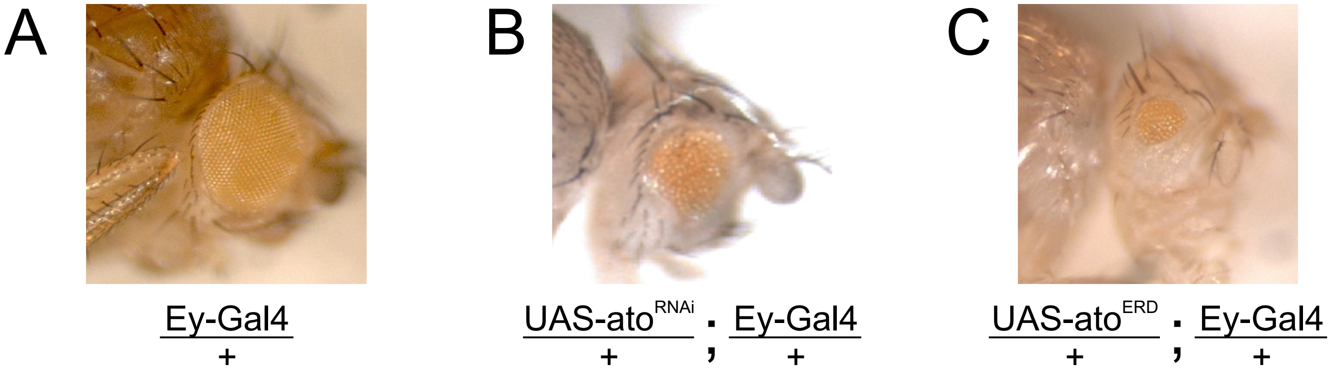


**Supplementary Figure 2: Adult loss of phenotypes of loss of *ato* function. A,** Representive picture *ey-Gal4*. **B,** Representive picture of *UAS-ato*$^{RNAi}$ driven by *ey-Gal4*. ato$^{RNAi}$ construct is active since the expression in the developing eye leads to a decrease in eye size. **C,** representative picture of *UAS-ato*$^{ERD}$ driven by *ey-Gal4*. The *ato*$^{ERD}$ leads to a phenocopy of the loss of *ato*.
