# Supplementary material for: The Atonal Proneural Transcription Factor Links Differentiation and Tumor Formation in Drosophila
Source: PLoS Biol. 2009 Feb 24;7(2):e1000040. doi: 10.1371/journal.pbio.1000040 (PMC2652389; doi:10.1371/journal.pbio.1000040)
Supplement: Figure S3 — Expression of ato in wild-type flies up-regulates Dap and phosphorylated JNK. Third instar eye disc from eyeless-Gal4/UAS-ato is shown. (2.06 MB PDF) [file pbio.1000040.sg003.pdf]

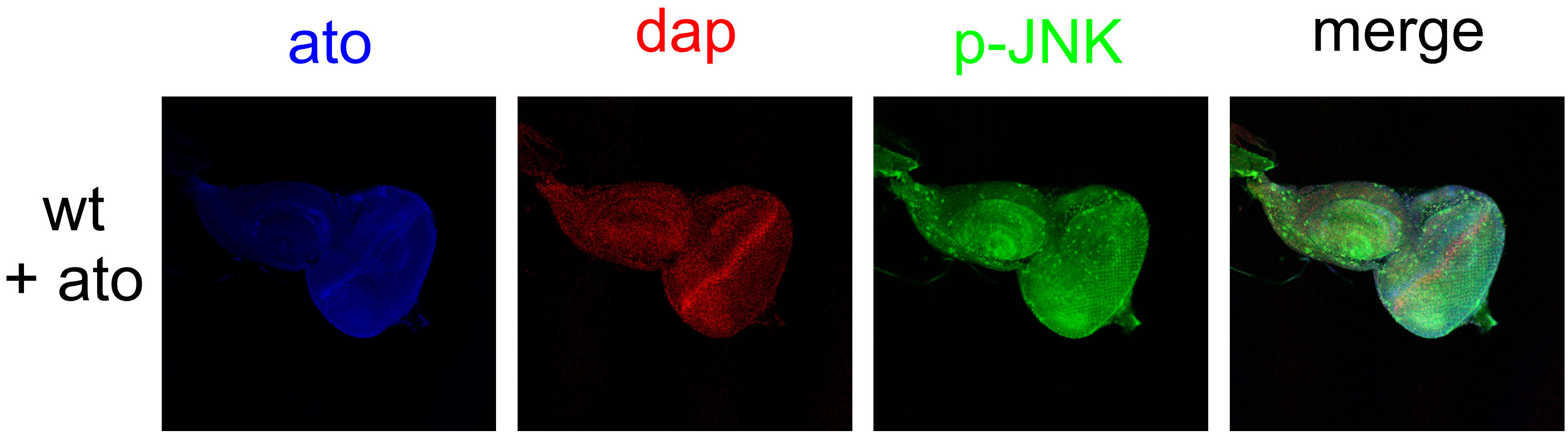

Supplementary Figure 3: Ato, dap qnd pJNK expression in WT eye discs. Expression of ato in wildtype flies, upregulates Dap and phosphorylated JNK. Third instar eye disc from eyeless-Gal4/UAS-ato
